# Cancer-Associated Fibroblasts Establish Spatially Distinct Prognostic Niches in Subcutaneous Colorectal Cancer Mouse Model

**DOI:** 10.3390/cancers17142402

**Published:** 2025-07-19

**Authors:** Zhixian Lin, Jinmeng Wang, Yixin Ma, Yanan Zhu, Yuhan Li, Zhengtao Xiao, Wei Zhao

**Affiliations:** 1 Department of General Surgery, The First Affiliated Hospital of Xi’an Jiaotong University, Xi’an 710061, China; 2 Institute of Molecular and Translational Medicine, Department of Biochemistry and Molecular Biology, Xi’an Jiaotong University Health Science Center, Xi’an 710061, China

**Keywords:** colorectal cancer, syngeneic tumor model, spatial heterogeneity, tumor microenvironment, metabolic–immune crosstalk

## Abstract

**Background/Objectives**: Subcutaneous tumor models are widely used in colorectal cancer (CRC) research due to their experimental accessibility; however, the spatial organization and regulatory mechanisms of their tumor microenvironment remain poorly understood.** Methods**: Here, we applied spatial transcriptomics to systematically characterize spatial heterogeneity within MC38 subcutaneous tumors in a syngeneic mouse model. **Results**: We identified two spatially distinct tumor zones, partitioned by cancer-associated fibroblasts (CAFs), that differ markedly in cellular composition, oncogenic signaling, immune infiltration, and metabolic states. One zone exhibited features of TGF-β-driven extracellular matrix remodeling, immune exclusion, and hyperproliferative metabolism, while the other was enriched for immunosuppressive macrophages, metabolic reprogramming via PPAR and AMPK pathways, and high-risk cell populations. Spatially resolved cell–cell communication networks further revealed zone-specific ligand–receptor interactions—such as ANGPTL4–SDC2 and PROS1–AXL—that underpin stromal remodeling and immune evasion and are associated with patient prognosis.** Conclusions**: Collectively, our study uncovers how region-specific cellular ecosystems and intercellular crosstalk establish prognostically divergent niches within subcutaneous CRC tumors, offering insights into spatially guided therapeutic strategies.

## Simple Summary

While subcutaneous colorectal cancer (CRC) models are commonly employed in biological and preclinical studies, their cell morphological organization and molecular landscape are not yet fully understood. Using spatial transcriptomics, we identified two spatially distinct tumor regions demarcated by cancer-associated fibroblasts (CAFs), each region exhibiting unique molecular signatures and immune niche. Moreover, we uncovered specific cancer–stroma cell interactions that actively remodel the tumor microenvironment to facilitate immune evasion. These findings uncover the previously unrecognized spatial heterogeneity of the CRC mouse model and highlight the critical role of specialized tumor niches in driving tumor development and progression.

## 1. Introduction

Colorectal cancer (CRC) is the second most prevalent cancer in China. In 2022, there were 517,100 new cases and 240,000 deaths, with mortality ranking fourth among all cancers [1]. Tumor heterogeneity, involving multiple factors such as genetic and epigenetic variations, represents a crucial mechanism underlying CRC drug resistance [2,3,4]. Understanding CRC heterogeneity is essential for precision medicine, yet existing preclinical models fall short. Immunodeficient patient-derived xenograft (PDX) models cannot accurately mimic dynamic immune microenvironments. Orthotopic models, despite their clinical relevance, are technically complex for high-throughput mechanistic studies [5,6]. In contrast, subcutaneous tumor models offer advantages such as visual monitoring and high-throughput screening [7,8]. For instance, these models have successfully revealed the tumor-suppressive effects of splicing factor SNRPB silencing and the synergistic therapeutic efficacy of YY2 overexpression combined with PD-1 inhibitors [9,10]. While most studies focus on gene targeting or drug screening, systematic analysis of the unique spatial architecture of the subcutaneous tumor microenvironment (TME) and its regulatory mechanisms on cancer cell behavior remains lacking.

Notably, significant differences exist between the TME of subcutaneous models and that of primary lesions, potentially limiting the clinical translatability of findings derived from these models [11,12]. Firstly, subcutaneous models exhibit fundamentally distinct immune cell infiltration patterns and treatment response characteristics compared to orthotopic models. For example, spatial distribution differences of CD4^+^/CD8^+^ T cells can significantly alter responses to radiotherapy and immune checkpoint inhibitor [13,14]. Secondly, subcutaneous tumors generally demonstrate slower growth rates compared to their orthotopic counterparts [13,15]. More importantly, experimental observations revealed distinct dominant subclones in subcutaneous tumors across mouse models with different immune statuses [16]. This immune pressure-driven clonal heterogeneity suggests that subcutaneous microenvironments may accelerate tumor adaptive evolution, consequently affecting therapeutic resistance evaluation and metastatic potential assessment. These limitations indicate substantial gaps in our understanding of subcutaneous model heterogeneity and underscore the need for deeper investigation into cancer cell state composition, functional differentiation, and niche formation mechanisms in these models.

Spatial transcriptomics (ST) is transforming cancer research by enabling in situ detection of molecular activity within cells and the inference of interactions between adjacent cells. This approach facilitates the identification of prognostic markers and links tissue architecture with patient outcomes [17,18]. ST also enables the spatial correlation of morphological patterns in tumor tissues—such as cancer cells, stroma, and non-tumoral regions—with their corresponding transcriptomic profiles, demonstrating significant potential for the characterization of CRC [19]. This study utilized spatial transcriptomic profiling to establish the spatial heterogeneity landscape of MC38 subcutaneous syngeneic tumors and identify two functionally distinct tumor zones. We identified the activation of zone-specific signaling pathways, functional polarization of stromal cells, and coordinated metabolic–immune network interactions as core drivers of spatial heterogeneity. Cancer B zone specifically was enriched with Scissor+ high-risk cell subpopulations, whose poor prognostic association originated from unique cell communication networks. These findings not only reveal the driving mechanisms of spatial differentiation in subcutaneous TME but also highlight the potential value of zone-specific therapeutic strategies in overcoming CRC heterogeneity-mediated drug resistance.

## 2. Results

### 2.1. Cell Organization and Histological Characteristics of the Murine Colorectal Cancer Model

**Figure 1:**
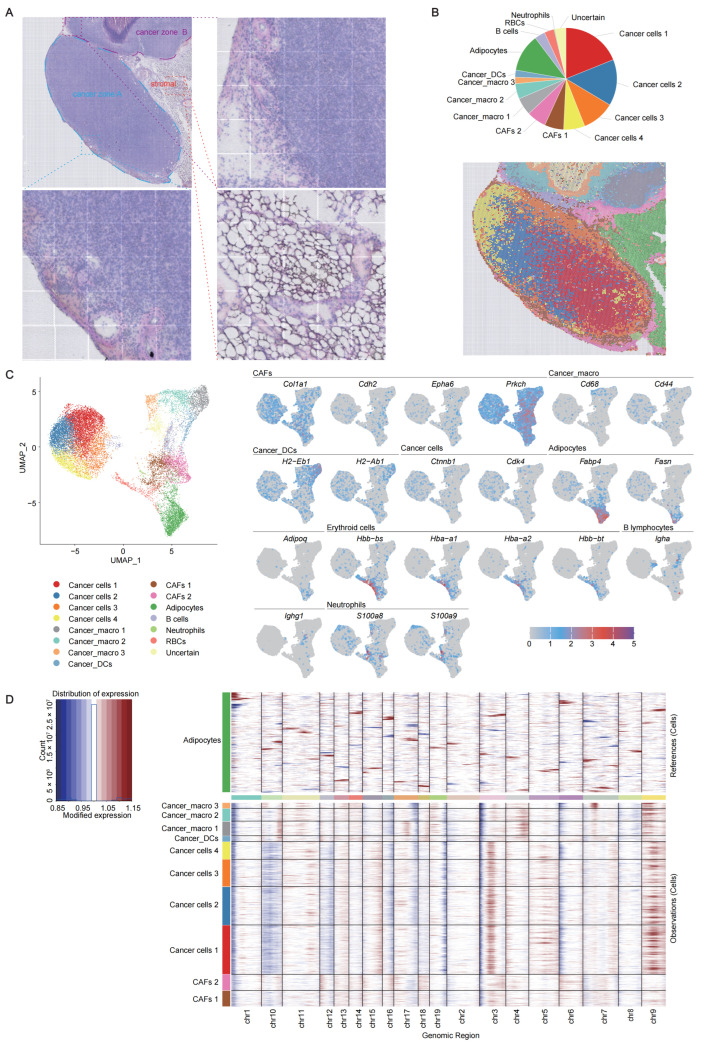
Cell type identification in MC38 mouse colorectal cancer (CRC) using spatial transcriptomics. (**A**) H&E-stained sections and localized views of MC38 CRC tissue. Based on histological features, the section was divided into three regions: cancer zone A, cancer zone B, and stromal zone, which are outlined with dashed lines. (**B**) Proportional distribution and spatial mapping of major cell types. Colors represent the different clusters. (**C**) UMAP projection of cell clusters (left) and marker gene expression patterns (right). In the left panel, colors represent different clusters, while in the right panel, colors indicate the expression levels of the corresponding genes. (**D**) Copy number variation (CNV) analysis of cancer zones using inferCNV. The *x*-axis represents different chromosomes, and the *y*-axis represents different clusters. Adipocytes serve as the reference, while the remaining clusters are considered observations.

To investigate the spatial heterogeneity of the CRC microenvironment, we performed spatial transcriptomic profiling on a tumor sample derived from the subcutaneous MC38 CRC model. On average, sequencing yielded 233 million reads per sample, with a median sequencing saturation of 64.41%. A total of 18,097 spatial spots with a diameter of 42 μm were captured and each spot detected approximately 860 genes and 1500 unique molecular identifiers (UMIs). After filtering out low-quality data—specifically, spots with fewer than three detected features and features expressed in fewer than 30 spots—we retained 18,095 high-quality spots for downstream analysis. Hematoxylin and eosin (H&E) staining enabled us to delineate three histologically distinct zones within the tumor sections (Figure 1A). Cancer zone A was characterized by enlargement and hyperchromatic nuclei, increased nuclear-to-cytoplasmic ratio, and relatively uniform cellular morphology. Neoangiogenesis was observed along the tumor margins of this zone. Cancer zone B shared similar nuclear features and peripheral signs of angiogenesis, but displayed localized differences in chromatin staining intensity, suggesting the presence of potential necrotic regions. The stromal zone showed low cellular density, elongated nuclei, foamy cytoplasm, and notable vascular and lymphocytic infiltration within the interstitial space.

Spatial spots were subsequently clustered into eight distinct categories and were annotated based on canonical gene expression profiles (Figure 1B,C and Figure S1A). Cancer cells, identified by the expression of *Ctnnb1* and *Cdk4*, were further stratified into four subtypes (cancer cells 1–4). These cancer cell-enriched regions exhibited copy number gains on chromosomes 3 and 9, and losses on chromosome 10 (Figure 1D). Cancer-associated fibroblasts (CAFs), marked by *Col1a1*, *Cdh2*, *Epha6*, and *Prkch*, were classified into two subtypes (CAFs 1 and 2).

Additionally, mixed cellular populations were detected, including a subset expressing both copy number gains and dendritic cell markers (*H2-Ab1*, *H2-Eb1*), annotated as cancer_dendritic cells (cancer_DCs). Another hybrid population co-expressing copy number gains and macrophage markers (*Cd68*, *Cd44*) was defined as cancer_macro and subdivided into three distinct subpopulations (cancer_macros 1–3). Notably, cancer_macro 1 showed pronounced copy number gains on chromosomes 4 and 17, whereas cancer_macro 2 and cancer_macro 3 exhibited gains on chromosomes 4, 7, and 10, respectively (Figure 1D).

Other TME components were identified using established markers: adipocytes (*Fabp4*, *Fasn*, *Adipoq*), erythroid cells (*Hbb-bs*, *Hba-a1*, *Hba-a2*, *Hbb-bt*), B lymphocytes (*Igha*, *Ighg1*), and neutrophils (*S100a8*, *S100a9*) (Figure 1C). Spatial mapping of marker gene expression revealed that cancer zone A was predominantly composed of cancer cell subtypes 1–4 and CAFs 1, while cancer zone B primarily contained cancer_macro subpopulations, cancer_DCs, and CAFs 2 (Figure 1B). These findings highlight the cellular and molecular heterogeneity within the subcutaneous tumor model, as well as the complexity of the tumor immune microenvironment potentially resulting from diverse tumor cell states and their interactions.

### 2.2. Molecular and Metabolic Differences Define Prognostically Distinct Cancer Zones

**Figure 2:**
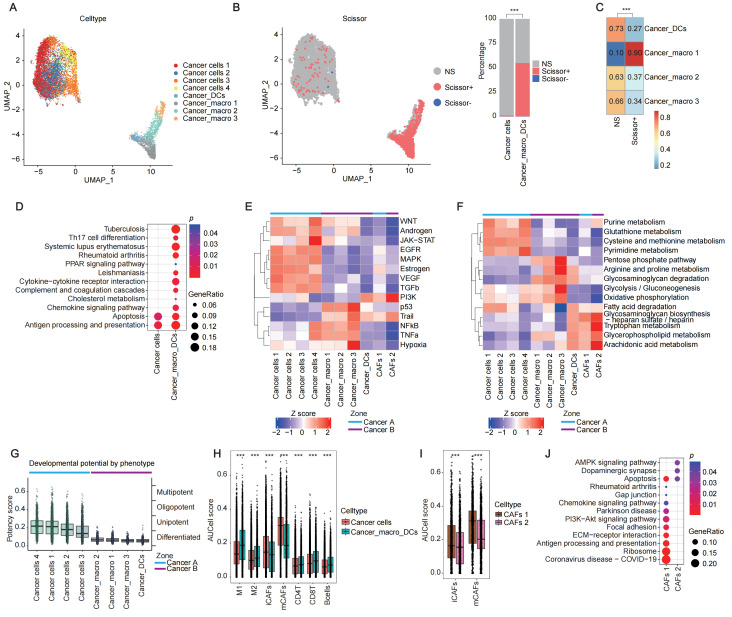
Functional divergence between cancer A and B zones. (**A**) UMAP projection of selected cell clusters. Colors represent different clusters. (**B**) Distribution of phenotype-associated spots in cancer A and B zones (*** *p* < 0.001; significance threshold = 0.05, Fisher’s exact test). Colors represent different Scissor outcomes. In the right panel, the *x*-axis denotes different cell clusters, and the *y*-axis indicates the proportion of each Scissor result within the clusters. (**C**) Intratumoral heterogeneity of phenotype-associated spots in the cancer B zone (*** *p* < 0.001; significance threshold = 0.05, Fisher’s exact test). The *x*-axis represents different Scissor outcomes, the *y*-axis represents different clusters, and the colors indicate the distribution proportions of each Scissor result within the clusters. (**D**) KEGG pathway enrichment of upregulated DEGs in cancer cells and cancer_macro_DCs (avg_log_2_FC > 0.25, *p* < 0.05). The *x*-axis represents different clusters, the *y*-axis represents different pathways, dot size indicates the GeneRatio, and color represents the *p*-value. (**E**) Activity scores of tumor-associated signaling pathways in cancer clusters. The *x*-axis represents different clusters, and the *y*-axis represents different pathways. The color of each square indicates the activity score, while the color of the lines denotes the zone in which the corresponding cluster is located. (**F**) Enrichment scores of metabolic pathways in cancer clusters. The *x*-axis represents different clusters, and the *y*-axis represents different pathways. The color of each square indicates the activity score, while the color of the lines denotes the zone in which the corresponding cluster is located. (**G**) Potency scores across distinct cell types. The *x*-axis represents different clusters, and the *y*-axis is divided into two parts: the left side shows the potency scores, and the right side indicates the corresponding potency levels. The color of the boxplots represents the potency score, while the color of the lines denotes the zone in which the corresponding cluster is located. (**H**) AUCell scores of immune cell gene signatures in cancer cells and cancer_macro_DCs (*** *p* < 0.001; significance threshold = 0.05, Wilcoxon rank-sum test). The *x*-axis represents different immune cell types, and the *y*-axis represents the AUCell scores. The colors of the boxplots indicate different clusters. (**I**) mCAFs and iCAFs signature scores in CAFs 1 and CAFs 2 (*** *p* < 0.001; significance threshold = 0.05, Wilcoxon rank-sum test). The *x*-axis represents different CAF types, and the *y*-axis represents the AUCell scores. The colors of the boxplots indicate different clusters. (**J**) KEGG pathway enrichment of upregulated DEGs between CAFs1 and CAFs2 (avg_log_2_FC > 0.25, *p* < 0.05). The *x*-axis represents different clusters, the *y*-axis represents different pathways, dot size indicates the GeneRatio, and the color represents the *p*-value.

To explore the clinical relevance of our findings, we integrated our data with TCGA CRC bulk sequencing data (377 samples after quality control) using the R package *Scissor *(v2.0.0). By analyzing both gene expression profiles and corresponding clinical information, we successfully identified 1539 phenotype-associated spots, the vast majority (1537) being Scissor+ spots associated with poor prognosis, which predominantly clustered in cancer zone B (Figure 2A,B). Only two Scissor− spots were detected, both confined to cancer zone A (Figure 2A,B). We merged cancer cells 1, 2, 3, and 4 in cancer zone A as cancer cells, and combined cancer_macros 1, 2, 3 and cancer_DCs in cancer zone B as cancer_macro_DCs. Fisher’s exact test was performed to analyze the distribution of Scissor+, Scissor−, and non-significant (NS) spots across selected cell types. The proportion of Scissor+ spots was significantly higher in cancer_macro_DCs compared to cancer cells, with cancer_macro 1 showing a notably higher proportion than cancer_macro 2, cancer_macro 3, and cancer_DCs (Figure 2B,C). Specifically, 90% of cancer_macro 1 spots were classified as Scissor+.

To further characterize spatial heterogeneity among cancer cells, we performed comparative gene expression analysis between cancer cells in cancer zone A and cancer_macro_DCs in cancer zone B. Differential expression analysis (log_2_FC > 0.25, *p* < 0.05), followed by KEGG pathway enrichment, revealed distinct functional patterns. Cancer cells in zone A showed significant activation of antigen processing and presentation and apoptosis pathways, whereas cancer_macro_DCs in cancer zone B exhibited elevated activity in complement and coagulation cascades, cytokine–cytokine receptor interactions, PPAR signaling, chemokine signaling, and cholesterol metabolism (Figure 2D), highlighting spatially distinct functional programs among cell types.

We employed the PROGENy algorithm to systematically quantify the activity of canonical oncogenic signaling pathways in the two cancer zones. Cancer zone A exhibited elevated activity in the WNT, JAK-STAT, EGFR, MAPK, VEGF, and TGFβ signaling pathways, whereas cancer zone B was characterized by predominant activation of the WNT, p53, TRAIL, NF-κB, TNFα, and hypoxia pathways (Figure 2E). Metabolic characterization further highlighted the functional distinctions between the two zones. Cancer cells in zone A exhibited enhanced nucleotide synthesis capacity, particularly in purine metabolism, glutathione metabolism, cysteine and methionine metabolism, and pyrimidine metabolism. Conversely, cancer_macro_DCs in cancer zone B demonstrated superior metabolic engagement in energy-producing and immunomodulatory pathways, including pentose phosphate–glycolysis coupling, oxidative phosphorylation, glycosaminoglycan degradation, lipid processing, and arginine-proline metabolism (Figure 2F). Finally, potency scoring analysis revealed that cancer zone A collectively possesses higher differentiation potential compared to cancer zone B (Figure 2G).

**Figure 3:**
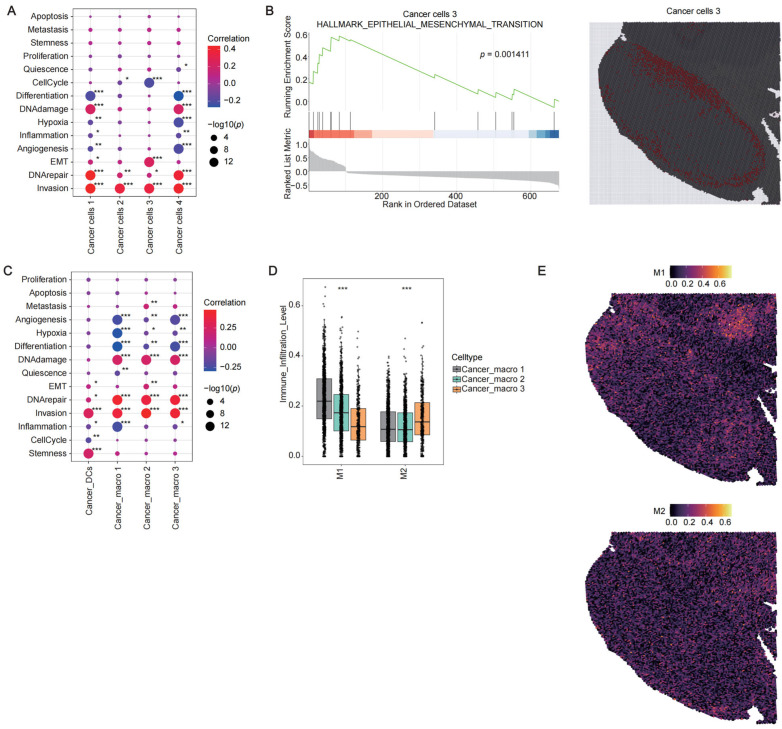
Tumor cell heterogeneity in cancer A and B zones (**A**) CancerSEA functional annotation of upregulated DEGs in cancer cells 1–4 (avg_log2FC > 0.25, *p* < 0.05, * *p* < 0.05, ** *p* < 0.01, *** *p* < 0.001). In the dot plot, the *x*-axis represents different clusters, and the *y*-axis represents different pathways. Dot size indicates −log_10_(*p*), and color represents the correlation. (**B**) GSEA enrichment of the EMT pathway in cancer cells 3 and spatial mapping of cancer cells 3. (**C**) CancerSEA functional annotation of upregulated DEGs in cancer_macros 1–3 and cancer_DCs (avg_log2FC > 0.25, *p* < 0.05, * *p* < 0.05, ** *p* < 0.01, *** *p* < 0.001). In the dot plot, the *x*-axis represents different clusters, and the *y*-axis represents different pathways. Dot size indicates −log_10_(*p*), and color represents the correlation. (**D**) AUCell scores of M1/M2 macrophage polarization markers (*** *p* < 0.001; significance threshold = 0.05, Kruskal–Wallis test). The *x*-axis represents different macrophage types, and the *y*-axis represents the AUCell scores. The colors of the boxplots indicate different clusters. (**E**) Spatial mapping of M1 and M2 scores. The colors represent the AUCell scores.

To further characterize stromal and immune cell infiltration in these zones, we calculated AUCell scores of different cell types using a curated marker gene list (Table S1). The analysis showed that cancer zone A had higher infiltration of myofibroblastic CAFs (mCAFs) and inflammatory CAFs (iCAFs). In contrast, cancer zone B exhibited elevated scores for M1 and M2 macrophages, CD4^+^ T cells, CD8^+^ T cells, and B cells, indicating more extensive immune infiltration (Figure 2H and Figure 3E). Notably, CAF subpopulations localized in different tumor zones also displayed functional divergence. CAFs 1 had remarkably higher mCAF and iCAF activity compared to CAFs 2 (Figure 2I). KEGG pathway analysis revealed that genes upregulated in CAFs from zone A were significantly enriched in the extracellular matrix (ECM)-receptor interaction and PI3K-Akt signaling pathway, consistent with their role in ECM remodeling. In contrast, CAFs in cancer zone B showed prominent enrichment in the AMPK signaling pathway, indicating the metabolic reprogramming toward immunosuppressive support (Figure 2J). Collectively, these findings highlight that spatially distinct tumor niches arising from a common origin can acquire divergent immune and metabolic characteristics. Cancer zone A reflects an immune-excluded microenvironment and hyperproliferative metabolism. In contrast, cancer zone B is distinguished by immunosuppressive immune infiltration and PPAR-mediated metabolic reprogramming.

### 2.3. Heterogeneity of Intratumoral Niches Across Distinct Cancer Zones

To delineate intratumoral heterogeneity, we performed gene set enrichment analysis (GSEA) using the CancerSEA database to assess functional states of cancer cells in distinct clusters within cancer zones A and B. In cancer zone A, four functionally specialized cancer cell clusters exhibited distinct molecular signatures and spatial distribution patterns (Figure 3A). Cancer cells 1 and 4 demonstrated similar functional polarization through their upregulated differentially expressed genes (DEGs), which were associated with high DNA damage, DNA repair, invasion, and low angiogenesis, hypoxia, and inflammation. Cancer cells 2 displayed low cell cycle activity. Notably, cancer cells 3 displayed DEGs significantly correlated with high epithelial-mesenchymal transition (EMT), invasion, and reduced cell cycle activity. These cells were spatially localized at the periphery of cancer zone A, adjacent to CAFs 1, suggesting potential involvement in immune niches regulation through stromal–tumor crosstalk (Figure 3A,B).

In cancer zone B, highly expressed DEGs across cancer_macro 1–3 subpopulations displayed shared characteristics, including enhanced invasion and reduced angiogenesis, hypoxia, and differentiation (Figure 3C). Each subpopulation exhibited unique molecular features: both cancer_macro 1 and 3 showed significantly suppressed inflammatory signatures, indicative of immunosuppressive states. Spatially, cancer_macro 1 was localized to the periphery of cancer zone B, surrounded by cancer_DCs, while cancer_macro 3 occupied the inner core of cancer zone B (Figure 1B). Cancer_macro 2 demonstrated strong associations with EMT and metastasis enhancement, positioned between cancer_macro 3 and B cells at the cancer zone B periphery. Additionally, cancer_DCs exhibited DEGs linked to elevated EMT, invasion, stemness, and diminished inflammation (Figure 3C). These spatially distinct cancer_macro 1–3 subpopulations and cancer_DCs collectively shaped heterogeneous immune niches.

Cancer_macros 1–3 overall demonstrated a predominance of M1-like phenotypes, although each of them exhibited distinct polarization characteristics. AUCell scoring analysis (*p* < 0.001) revealed that both cancer_macro 1 and 2 demonstrated strong M1 features, whereas cancer_macro 3 displayed prominent M2 characteristics. Notably, the M1 score of cancer_macro 1 was significantly higher than that of other clusters (Figure 3D,E). In summary, distinct tumor microecological landscapes were observed across spatial zones. Specifically, the peripheral region of cancer zone A was predominantly composed of cancer cell cluster 3, which displayed elevated EMT signatures, suggesting its high invasiveness at the tumor frontiers. In contrast, cancer zone B exhibited heterogeneity in macrophage polarization across cancer_macro clusters 1–3, likely reflecting distinct, compartmentalized immune regulatory mechanisms within the tumor microenvironment.

### 2.4. Zone-Specific Cell–Cell Interactions Define Distinct Tumor Niches Associated with Patient Prognosis

**Figure 4:**
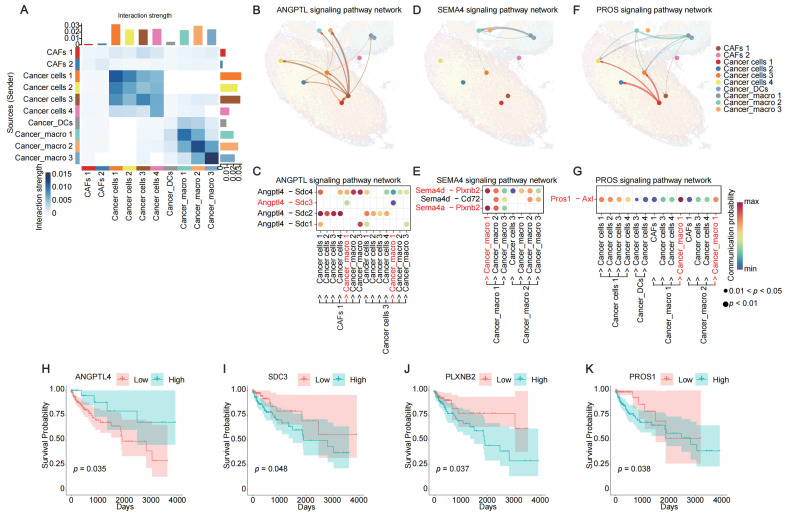
Cell–cell interaction networks in cancer A and B zones. (**A**) Interaction strength in cancer A and B zones. The x- and y-axes represent different cell types. The color of each square indicates the interaction strength between clusters. The height of the bars reflects the magnitude of these interactions, while the color of the bars represents the corresponding clusters. (**B**) Spatial mapping of ANGPTL communication pathways. The color of each dot represents a different cluster, and the direction of the arrows indicates the flow of communication from ligand-expressing to receptor-expressing clusters. (**C**) Enriched ligand–receptor pairs in ANGPTL communication pathways. The *x*-axis represents the direction of communication ligand-expressing to receptor-expressing clusters, and the *y*-axis shows different ligand–receptor pairs. Dot color indicates the communication probability, while dot size represents the *p*-value. (**D**) Spatial mapping of SEMA4 communication pathways. The color of each dot represents a different cluster, and the direction of the arrows indicates the flow of communication from ligand-expressing to receptor-expressing clusters. (**E**) Enriched ligand-receptor pairs in SEMA4 communication pathways. The *x*-axis represents the direction of communication ligand-expressing to receptor-expressing clusters, and the *y*-axis shows different ligand–receptor pairs. Dot color indicates the communication probability, while dot size represents the *p*-value. (**F**) Spatial mapping of PROS communication pathways. The color of each dot represents a different cluster, and the direction of the arrows indicates the flow of communication from ligand-expressing to receptor-expressing clusters. (**G**) Enriched ligand–receptor pairs in PROS communication pathways. The *x*-axis represents the direction of communication ligand-expressing to receptor-expressing clusters, and the *y*-axis shows different ligand–receptor pairs. Dot color indicates the communication probability, while dot size represents the *p*-value. (**H**) Survival analysis of ANGPTL4 in TCGA CRC cohorts (significance threshold = 0.05). The *x*-axis represents survival time, and the *y*-axis represents survival probability. Different colors indicate groups with different levels of gene expression. (**I**) Survival analysis of SDC3 in TCGA CRC cohorts (significance threshold = 0.05). The *x*-axis represents survival time, and the *y*-axis represents survival probability. Different colors indicate groups with different levels of gene expression. (**J**) Survival analysis of PLXNB2 in TCGA CRC cohorts (significance threshold = 0.05). The *x*-axis represents survival time, and the *y*-axis represents survival probability. Different colors indicate groups with different levels of gene expression. (**K**) Survival analysis of PROS1 in TCGA CRC cohorts (significance threshold = 0.05). The *x*-axis represents survival time, and the *y*-axis represents survival probability. Different colors indicate groups with different levels of gene expression.

To elucidate the causes of distinct TME features, we performed spatial cell–cell interaction analysis. The results revealed that both cancer zone A and cancer zone B exhibited pronounced spatial proximity-dependent communication patterns (Figure 4A and Table S2). Despite originating from the same injection site, these two regions developed distinct microenvironmental ecosystems. Ligand–receptor interaction networks quantified through the CellChat system demonstrated that cancer zone A was characterized by COLLAGEN, FN1, and ANGPTL signaling pathways (Figure 4B and Figure S2A,C). In contrast, cancer zone B specifically activated the SEMA4 pathway (Figure 4D). The PROS signaling pathway was active in both zones (Figure 4F).

In cancer zone A, cancer cells primarily communicated through the COLLAGEN pathway via ligand–receptor interactions, including *Col1a1-(Itga9+Itgb1)*, *Col1a1-Cd44*, *Col1a2-(Itga9+Itgb1)*, and *Col1a2-Cd44*, with *Col1a2-(Itga9+Itgb1)/Cd44* showing the strongest interaction strength (Figure S2B). Within the FN1 pathway, *Fn1-Cd44*, *Fn1-Sdc1*, and *Fn1-Sdc4* mediated intercellular communication among cancer cells, with *Fn1-Cd44* being the most potent ligand–receptor pair (Figure S2D). The ANGPTL4 pathway operated through *Angptl4-Sdc2* and *Angptl4-Sdc4* interactions, where *Angptl4-Sdc2* predominantly facilitated specific communication between CAFs 1 and cancer cells (Figure 4C).

In cancer zone B, the SEMA4 pathway relied on *Sema4a-Plxnb2*, *Sema4d-Cd72*, and *Sema4d-Plxnb2* interactions, with *Sema4a/d-Plxnb2* showing particularly strong communication between cancer_macro 1 cells (Figure 4E). The PROS pathway exhibited prominent *Pros1-Axl* interactions, most significant between cancer_macro 1/2 and cancer_macro 1 cells (Figure 4G).

Survival analysis, following human homolog conversion of murine genes, revealed that high expression of the *ANGPTL4* ligand correlated significantly with favorable prognosis (Figure 4H), whereas expression of the *SDC3* receptor, specifically enriched in cancer_macro 1 cells, was associated with poor prognosis (Figure 4C,I). Among cancer_macro 1 interactions, both *PROS1* ligand and *PLXNB2* receptor demonstrated significant associations with adverse clinical outcomes (Figure 4J,K).

Collectively, spatially distinct intercellular communication networks were observed between tumor microenvironmental zones. In cancer zone A, *Col1a2-Itga9/Itgb1* and *Fn1-Cd44* interactions among cancer cells, along with *Angptl4-Sdc2* signaling between CAFs 1 and cancer cells, collectively activated stromal remodeling and promoted EMT. Conversely, cancer zone B utilized *Sema4a/d-Plxnb2* and *Pros1-Axl* interactions to coordinate crosstalk within cancer_macro 1 populations, driving immune evasion and promoting tumor invasion and metastasis.

## 3. Discussion

The complexity and dynamic nature of the TME in CRC profoundly influences its progression and therapeutic resistance [20]. This study employed spatial transcriptomics to reveal the spatial heterogeneity of the TME in a syngeneic mouse model. We discovered that subcutaneous injected tumor cells formed distinct spatial regions, each developing unique immune microenvironments, including immune cell infiltration levels, functional states of immune cells, and cell–cell communication. Cancer zone A represented an immune-excluded (“cold”) tumor niche, while cancer zone B displayed higher infiltration but functionally suppressed immune states.

Within the TME, diverse cell populations in distinct functional states contribute to malignant progression through selective activation of signaling pathways and synergistic metabolic–immune networks. CAFs could be categorized into mCAFs and iCAFs. mCAFs function in ECM remodeling within the TME, while iCAFs promote oncogenesis by altering the TME [21,22,23]. Macrophages demonstrated a dual role, existing along a functional continuum between M1-like (pro-inflammatory, anti-tumoral) and M2-like (immunosuppressive, pro-angiogenic) phenotypes [24]. The high nucleotide metabolic activity in tumor cells of zone A suggests their increased metastatic potential [25]. In cancer zone A, CAFs 1 were recruited through the TGF-β signaling axis, promoting their differentiation into mCAFs. This led to excessive deposition and increased rigidity of ECM components, which not only restricted cancer cell migration and metastasis but also formed physical barriers limiting immune cell infiltration, thereby establishing an immunosuppressive niche [26,27,28]. Cancer zone B activates complement and coagulation cascades along with PPAR signaling pathways. This hyper-glycolytic environment may upregulate the HIF-1α pathway to induce PD-L1 expression. Cancer cells can also deplete microenvironmental arginine through arginine metabolism to suppress T cell function [29,30,31,32,33]. CAFs 2 regulate fatty acid degradation and tryptophan metabolism via the AMPK pathway, promoting M2 macrophage polarization and further reinforcing immunosuppression [34,35].

Region-specific intercellular communication networks created distinct tumor niches. In cancer zone A, cancer cells primarily engaged in *Col1a1/Col1a2*-*Itga9/Itgb* and *Fn1*-*Cd44* interactions to promote invasion and drive ECM remodeling [36,37,38]. CAFs 1 communicated with cancer cells 3 located at the cancer zone A periphery through *Angptl4*-*Sdc2* interactions, co-activating EMT and MAPK signaling pathways [39]. This subpopulation received microenvironmental stimuli distinct from core-region cancer cells, potentially promoting epithelial differentiation loss and ultimately manifesting high EMT marker expression [40,41].

In cancer zone B, a defining feature was the specific enrichment of Scissor+ high-risk cell subpopulations. Although different cell types in this zone demonstrated close and similar interactions, their expression levels in shared receptor–ligand complexes differed. For instance, cancer_macro 1 specifically expressed the *Sdc3* receptor gene. This subpopulation exhibited the highest communication probabilities in both *Pros1-Axl* and *Sema4a/d-Plxnb2* pathways. Notably, *PROS1* ligand and *SDC3/PLXNB2* receptors showed significant correlation with poor prognosis, potentially explaining why cancer_macro 1 represents the core immunosuppressive subpopulation in cancer zone B. Previous studies indicate that the PROS1-AXL axis fosters an immunosuppressive environment by downregulating MHC I–mediated antigen presentation and inhibiting NKG2D signaling [42,43,44]. Plexin-B2 restricts macrophage motility through negative regulation of Rac/Cdc42 signaling, potentially impairing antigen presentation and tumoricidal functions [45,46,47].

The spatial heterogeneity of subcutaneous CRC mouse models provides valuable insights for precision therapeutics. Immune-excluded regions characterized by dense ECM barriers hinder drug penetration. To overcome this, strategies such as targeting mCAFs to inhibit collagen biosynthesis or delivering collagenase locally via hydrogels to fibrotic areas may enhance therapeutic efficacy [48]. Immune-suppressive regions enriched with dysfunctional immune cells can be targeted by modulating arginine depletion, tryptophan metabolism, or signaling pathways such as PPAR and AMPK to reverse immunosuppression in core immune subpopulations and enhance therapeutic efficacy. Variations in stromal composition shapes the spatial distribution of immune cell lineages, thus significantly influencing the assessment of immune infiltration, patient prognosis, and responses to immunotherapy. High stromal content forms structural barriers that limit the infiltration of effector immune cells, such as T cells, into the tumor core, thereby leading to immune exclusion and suppression. This directly impacts the efficacy of immunotherapy and clinical outcomes of patients [49]. However, while widely used for their simplicity and controllability, subcutaneous models limit the simulation of metastasis and the native tissue microenvironment [50]. Therefore, integrating complementary models, specifically the Patient-Derived Orthotopic Xenograft (PDOX) model and PDX models, enables cross-validation of tumor–microenvironment interactions and therapeutic responses. This integration provides a more comprehensive understanding, thereby enhancing scientific rigor and clinical relevance. Moreover, there is substantial heterogeneity in the abundance, subset composition, and functional states of immune cells across different tumor regions, which is closely associated with tumor progression and therapeutic responses [51]. Therefore, systematic spatial profiling of cancer cells is fundamental to advancing our understanding of the tumor ecosystem and its underlying cellular interactions.

These results suggest that spatial heterogeneity in murine tumor models may influence experimental outcomes, emphasizing the necessity to consider intratumoral spatial variability in inter-group comparative analyses. However, this study has several limitations. First, the limited sample size restricts a comprehensive investigation of spatial heterogeneity across CRC tumor models in different murine systems. Second, region-specific ligand–receptor interactions between cancer cells and CAFs—such as ANGPTL4–SDC2, PROS1–AXL, and SEMA4A/D–PLXNB2—have not been systematically validated in large clinical cohorts, and their therapeutic relevance in clinical contexts remains to be determined.

In conclusion, by applying spatial transcriptomics to a subcutaneous CRC mouse model, we identified functionally distinct tumor niches defined by CAF-mediated structural organization, divergent cellular compositions, heterogeneous metabolic and immune profiles, and zone-specific intercellular communication networks. These spatial features shape local immune states and are associated with differential prognostic potential.

## 4. Method Details

### 4.1. Experimental Model Details

In this study, an 8-week-old C57BL/6 mouse (Chengdu Gembio Co., Ltd., Chengdu, China) was used. It was acclimated for 7 days before the experiment, kept in a 12 h light/dark cycle at a temperature of 22 °C, and provided with ad libitum food and water. The MC38 colorectal cancer cell line was used for tumor induction. Cells were cultured in DMEM containing 10% fetal bovine serum (FBS) at 37 °C in a 5% CO_2_ incubator, with the medium being changed every 2 days.

Prior to inoculation, cells were grown to the logarithmic phase, collected, and washed three times with phosphate-buffered saline (PBS). Each mouse received a single subcutaneous (s.c.) injection of 5 × 10^5^ MC38 cells into the shoulder zone of the back. The tumors were allowed to develop for 18 days post-inoculation, after which the mice were euthanized, and the tumor tissue was quickly excised. The excised tumor tissue was immediately embedded in optimum cutting temperature (OCT) compound (Sakura Finetek, Torrance, CA, USA; American Cherry Blossom OCT Frozen Slice Embedding Agent, Cat#: 4583) on dry ice and stored at −80 °C for subsequent histological analysis. All animal experiments were approved by the Biomedical Ethics Committee of Health Science Center of Xi’an Jiaotong University.

### 4.2. Spatial Transcriptomics Library Preparation, Sequencing, and Preprocessing

The surgically obtained mouse CRC tissue from a mouse was trimmed and tissue surfaces were quickly rinsed with a pre-cooled solution of PBS (RNase free) or normal saline to remove residual blood, and sterile gauze was used to blot the surface fluid. Small fragments of each tissue were snap-frozen in isopentane pre-chilled with liquid nitrogen and OCT compound. Then, the frozen tissue was cut in a pre-cooled cryostat at 10 μm thickness and systematically placed on chilled BMKMANU S1000 Tissue Optimization Slides and BMKMANU S1000 Gene Expression Slides and stored at −80 °C until use.

Reverse transcription (RT), second-strand cDNA synthesis, adaptor ligation, and a second RT were generated and libraries were constructed according to the performer’s protocol. Sequencing handles and indexes were added in an indexing PCR and the finished libraries were purified and quantified. Sequencing was performed on the Illumina NovaSeq 6000 with a sequencing depth of at least 50,000 reads per spot (100 μm) and 150 bp (PE150) paired-end reads (performed by Biomarker Technologies Corporation, Beijing, China). Primer spots were stained by hybridization of fluorescently labeled probes and imaged on the Metafer Slide Scanning platform (Pannoramic MIDI, 3DHISTECH Ltd., Budapest, Hungary). The resulting spot image was loaded into the BSTMatrix (v2.2) and BSTViewer (v2.2) along with the previously obtained BF tissue image of the same area. The two images were aligned and the built-in tissue recognition tool was used to extract spots covered by tissue. The L6 resolution level was ultimately selected for downstream analysis.

### 4.3. Differential Expression Profiling and Cellular Cluster Annotation

Dimensionality reduction and cluster identification were performed using Seurat (v4.4.0) [52]. Uniform Manifold Approximation and Projection (UMAP) was implemented for nonlinear dimensionality reduction to visualize cellular distributions in two-dimensional space. Cellular clusters were resolved via the following computational pipeline: (1) nearest-neighbor graph construction using the FindNeighbors function (Euclidean distance metric, k.param = 20), followed by (2) modularity optimization-based clustering via FindClusters (resolution = 0.8). Cluster-specific marker genes were identified using the FindAllMarkers algorithm, with differential expression thresholds set at avg_log_2_FC > 0.25 and *p* < 0.05. Cluster annotations were assigned through integrative evaluation of canonical marker expression profiles and copy number variation (CNV) scores derived from inferCNV analysis. Proportional representation of clusters was quantified and visualized using ggplot2 (v3.5.1). Spatial mapping of annotated cell types onto histopathological sections was achieved through the SpatialDimPlot function.

### 4.4. Functional Enrichment Analysis

Pathway enrichment analyses were systematically conducted for 12 spot subsets (cancer cells 1–4, cancer_macros 1–3, cancer_DCs, CAFs 1–2, Scissor+/Scissor−/NS groups) using clusterProfiler (v4.6.2) [53]. Upregulated DEGs (avg_log2FC > 0.25, *p* < 0.05) were subjected to Kyoto Encyclopedia of Genes and Genomes (KEGG) pathway analysis. Enrichment significance was graphically represented through node size (GeneRatio) and color gradient (*p*), with all displayed pathways meeting statistical thresholds (*p* < 0.05, *q* < 0.4). For cross-species translational analysis, human ortholog conversion was performed via the homologene package (v1.4.68.19.3.27) [54], followed by functional annotation using CancerSEA: https://ngdc.cncb.ac.cn/databasecommons/database/id/6092 (accessed on 7 March 2025). Pathway correlation networks were visualized using ggplot2, with edge colors denoting negative (blue) or positive (red) associations (* *p* < 0.05, ** *p* < 0.01, *** *p* < 0.001) and size denoting correlation.

### 4.5. Immune–Stromal Microenvironment Quantification

Cellular infiltration dynamics were characterized through AUCell-based deconvolution using lineage-specific marker gene sets. Statistical validation of infiltration patterns was performed using nonparametric tests: the Wilcoxon rank-sum test for pairwise comparisons and the Kruskal–Wallis test for multi-group analyses, with significance thresholds defined as *p* < 0.05 (*** *p* < 0.001).

### 4.6. CNV Landscape Reconstruction

Somatic copy number alteration (CNA) profiles were independently resolved for cancer cells 1–4, cancer_macros 1–3, cancer_DCs, and CAFs 1–2 using inferCNV (v1.23.0) [55]. Critical parameters included signal cutoff = 0.1, group-based clustering (cluster_by_groups = TRUE), hidden Markov model-based smoothing (HMM = TRUE), and noise reduction (denoise = TRUE).

### 4.7. PROGENy Pathway Activity Inference

Spatially resolved pathway activation states were decoded using the murine PROGENy model (v1.20.0) [56], focusing on the top 500 pathway-responsive genes as per benchmark recommendations. Pathway activity scores were computed through linear regression modeling of gene expression signatures.

### 4.8. Metabolic Pathway Activity Quantification

Single-cell metabolic flux analysis was conducted using scMetabolism (v0.2.1) [57], employing the AUCell algorithm to score 79 conserved metabolic pathways. Metabolic heterogeneity across clusters was evaluated through comparative pathway activity profiling. For all presented pathways, the percentage of missing genes is less than 40%.

### 4.9. Pseudotemporal Trajectory Reconstruction

Differentiation dynamics were modeled using Monocle3 (v1.3.7) for pseudotemporal ordering [58]. Developmental trajectory validation was augmented by CytoTRACE2 (v1.1.0) [59], which estimates differentiation potential through gene expression entropy analysis.

### 4.10. Intercellular Communication Network Analysis

Ligand–receptor-mediated crosstalk among cancer cells 1–4, cancer_macros 1–3, cancer_DCs, and CAFs 1–2 was deciphered using CellChat (v2.1.2) [60]. Interaction networks were resolved through (1) netVisual_heatmap for global interaction strength mapping, (2) netVisual_bubble for significant ligand–receptor pair visualization (*p* < 0.05), and (3) netVisual_aggregate for spatial colocalization analysis of selected pathways.

### 4.11. Clinical Outcome Correlation Analysis

Survival associations were investigated in 377 TCGA colorectal adenocarcinoma (COAD) patients using data from https://www.cancer.gov/tcga (accessed on 19 March 2025) using survival (v3.5-3) and survminer (v0.5.0) packages [61,62]. Prognostic ligand and receptors were identified through maximally selected rank statistics via surv_cutpoint, with Kaplan–Meier curves generated using ggsurvplot (*p* < 0.05).

### 4.12. Spatio-Prognostic Zone Identification

The Scissor algorithm (v2.0.0) [63] was implemented to resolve spatial domains associated with clinical outcomes (Scissor+/Scissor−/NS groups) through integrative analysis of transcriptomic patterns and TCGA survival data after human ortholog conversion of expression matrix. When running the Scissor function, we set the parameters alpha = 0.0005 and family = “cox”. Proportional distribution significance was validated via Fisher’s exact test. Upregulated DEGs of both Scissor+ and Scissor−/NS groups were identified through differential expression analysis (avg_log_2_FC > 0.25, *p* < 0.05) comparing Scissor+ versus Scissor−/NS groups.

### 4.13. Quantification and Statistical Analysis

Statistical validation of AUCell scores was performed using nonparametric tests: the Wilcoxon rank-sum test for pairwise comparisons and the Kruskal–Wallis test for multi-group analyses, with significance thresholds defined as *p* < 0.05 (*** *p* < 0.001). Proportional distribution significance was validated via Fisher’s exact test.

## 5. Conclusions

Spatial transcriptomic analysis revealed that in a subcutaneous colorectal cancer (CRC) murine model, cancer-associated fibroblasts (CAFs) partition the tumor into functionally heterogeneous regional “niches”. These niches exhibit significant differences in cellular composition, metabolic states, and local intercellular communication networks. Such spatial features shape the local immune microenvironment, leading to the formation of immune-excluded “cold” tumor regions in some areas, while other regions display high levels of immune cell infiltration but with suppressed functional activity. Region-specific intercellular interactions drive the emergence of tumor niches with distinct prognostic outcomes, which are closely associated with clinical prognosis.

## Data Availability

The raw sequencing data reported in this study have been deposited in the Genome Sequence Archive under accession number PRJCA041767 and are publicly available at https://ngdc.cncb.ac.cn/gsa (accessed on 18 June 2025) [64,65]. Any additional information required to reanalyze the data reported in this paper is available from the lead contact upon request.

## Supplementary Materials

The following supporting information can be downloaded at https://www.mdpi.com/article/10.3390/cancers17142402/s1, Figure S1: Spatial mapping of marker genes; Figure S2: Communications in cancer zone A and cancer zone B; Table S1: The main markers of different cell types; Table S2: Important pathways and LR pairs in cancer zone A and cancer zone B.
