# Supplementary material for: Cancer-Associated Fibroblasts Establish Spatially Distinct Prognostic Niches in Subcutaneous Colorectal Cancer Mouse Model
Source: Cancers (Basel). 2025 Jul 19;17(14):2402. doi: 10.3390/cancers17142402 (PMC12293927; doi:10.3390/cancers17142402)
Supplement: Supplementary file 1 [file cancers-17-02402-s001.zip › cancers-3746144_Figure S1.pdf]

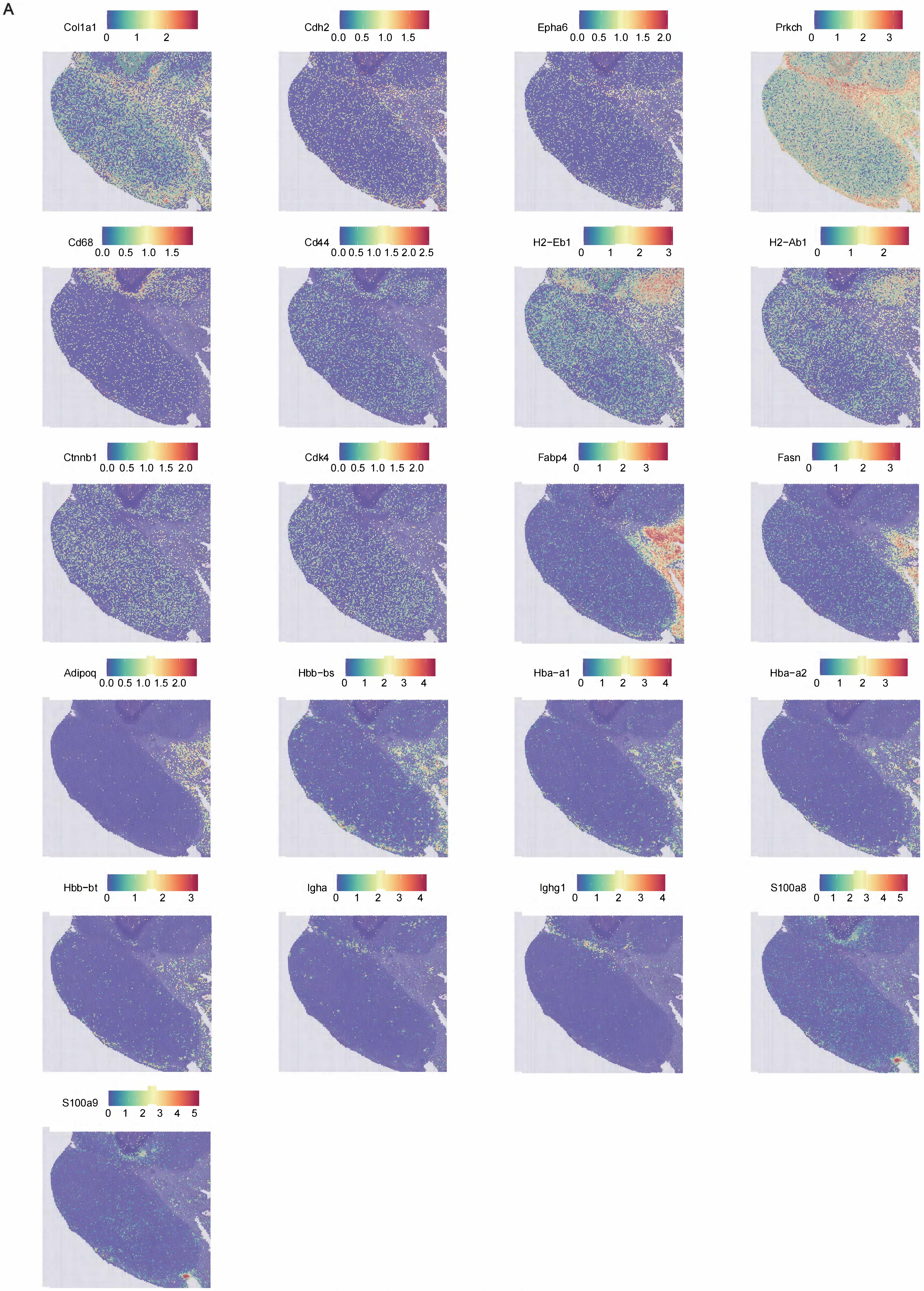

Supplementary Figure S1. Spatial mapping of marker genes.
(A) Spatial distribution of marker genes for each cluster overlaid on the H&E-stained tissue section
