# Supplementary material for: Cancer-Associated Fibroblasts Establish Spatially Distinct Prognostic Niches in Subcutaneous Colorectal Cancer Mouse Model
Source: Cancers (Basel). 2025 Jul 19;17(14):2402. doi: 10.3390/cancers17142402 (PMC12293927; doi:10.3390/cancers17142402)
Supplement: Supplementary file 1 [file cancers-17-02402-s001.zip › cancers-3746144_Figure S2.pdf]

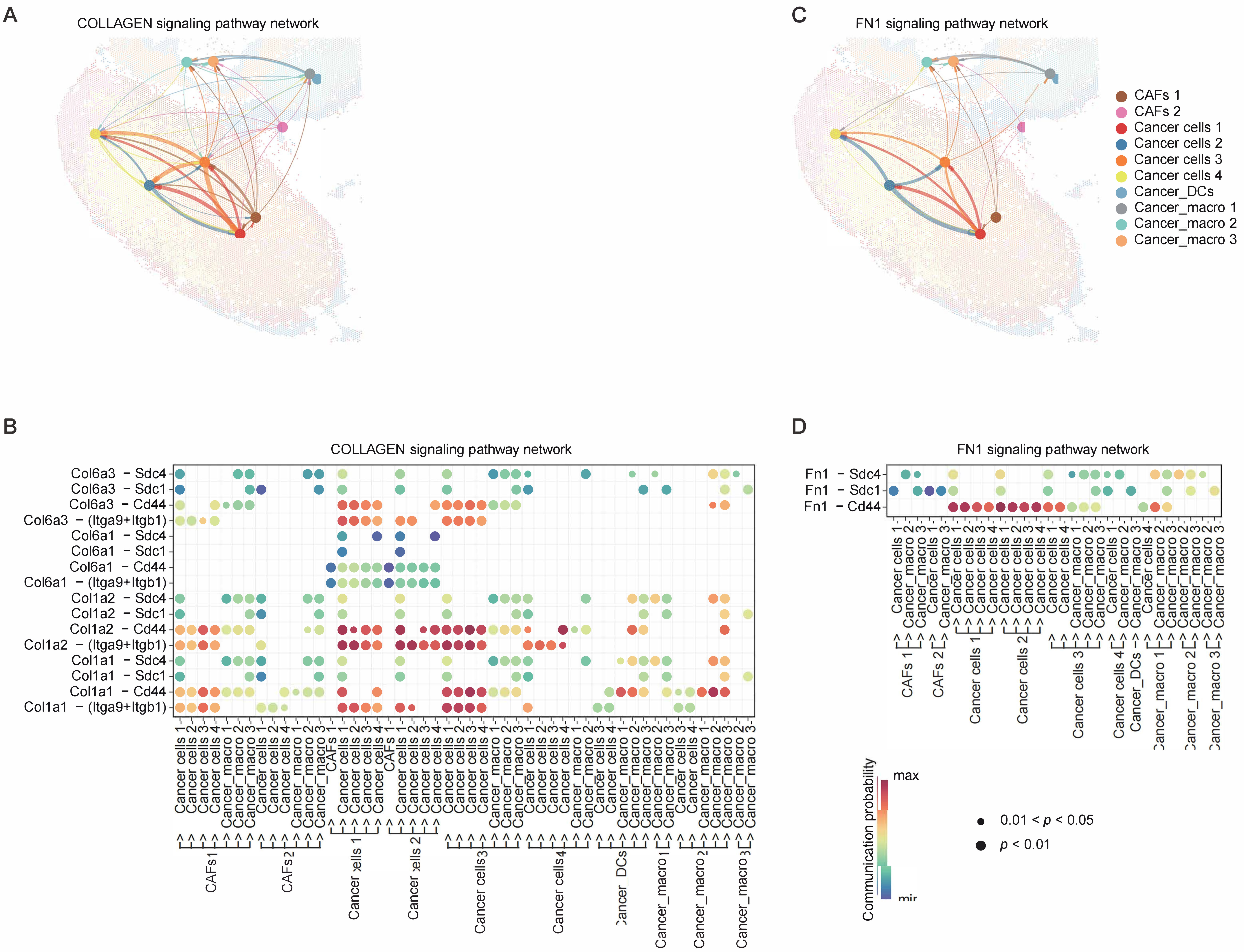

Supplementary Figure S2. Communications in Cancer A and Cancer B.
(A) Interaction strength of the COLLAGEN signaling pathway. The color of each dot represents a different cluster, and the direction of the arrows indicates the flow of communication from ligand-expressing to receptor-expressing clusters. (B) Interaction strength of ligand-receptor pairs in the COLLAGEN signaling pathway. The x-axis represents the direction of communication ligand-expressing to receptor-expressing clusters, and the y-axis shows different ligand-receptor pairs. Dot color indicates the communication probability, while dot size represents the p-value. (C) Interaction strength of the and FN1 signaling pathway. The color of each dot represents a different cluster, and the direction of the arrows indicates the flow of communication from ligand-expressing to receptor-expressing clusters. (D) Interaction strength of ligand-receptor pairs in the FN1 signaling pathway. The x-axis represents the direction of communication ligand-expressing to receptor-expressing clusters, and the y-axis shows different ligand-receptor pairs. Dot color indicates the communication probability, while dot size represents the p-value.
